# Impact of a Structured Educational Intervention on Primary Care Physicians’ Knowledge of Soft Tissue Sarcomas: A Multicenter Quasi-Experimental Study

**DOI:** 10.3390/healthcare14213621

**Published:** 2026-10-26

**Authors:** Fernando Leiva-Cepas, Ignacio Jimena Medina, Raquel Gracia-Rodríguez, Esperanza Romero-Rodriguez

**Affiliations:** 1 Pathology Unit, Department of Morphological and Sociosanitary Sciences, Faculty of Medicine and Nursing, University of Córdoba (UCO), 14071 Cordoba, Spain; 2 Histology Unit, Department of Morphological and Sociosanitary Sciences, Faculty of Medicine and Nursing, University of Córdoba (UCO), 14004 Cordoba, Spain; 3 Primary Health Care District of Córdoba-Guadalquivir, 14011 Cordoba, Spain; graciarodriguezraquel@gmail.com; 4 Primary Health Care District of Córdoba-Guadalquivir, Maimonides Institute for Biomedical Research (IMIBIC), Reina Sofía University Hospital, University of Córdoba (UCO), 14004 Cordoba, Spain

**Keywords:** sarcoma, primary care, medical education, diagnostic delay, referral pathways, guidelines, survey

## Abstract

**Highlights:**

**What are the main findings?**

- A multidisciplinary educational training focused on the symptoms, diagnosis, and treatment of soft tissue sarcomas improved the knowledge of primary care physicians.
- Participants reported limited prior training on tumors, representing significant educational gaps in the recognition of rare tumors in primary care settings. An improvement in knowledge was shown after the completion of the workshop given by a multidisciplinary team (primary care physician, oncologist and a pathologist).

**What are the implications of the main findings?**

- Specific educational training can improve the diagnosis, referral, and treatment of suspected sarcoma cases in primary care.
- Multidisciplinary training for different specialists could strengthen collaboration between primary care and hospital specialists, and improve adherence to referral guidelines.

**Abstract:**

Background: Soft tissue sarcomas (STS) are rare malignant tumors characterized by diagnostic complexity, low incidence, and frequent diagnostic delay. Primary care physicians are often the first professionals to assess patients with suspicious masses, yet important gaps in sarcoma-related knowledge have been reported. This study evaluated the impact of a multidisciplinary educational intervention on primary care physicians’ knowledge of STS. Methods: A multicenter single-group quasi-experimental pre-post study without a control group was conducted in Spain. Forty-two primary care physicians and Family and Community Medicine residents who voluntarily agreed to participate attended an in-person workshop delivered by a family physician, an oncologist, and a pathologist. Knowledge was assessed before and after the intervention using a structured questionnaire. Descriptive and inferential analyses were performed, including correction for multiple comparisons. Results: Forty-two participants completed both assessments. Self-perceived knowledge improved consistently across all 36 items, and these differences remained significant after correction for multiple comparisons (all *p*-adj < 0.005). Effect sizes were large overall (r = 0.48–0.84), and in most domains more than 80% of participants showed improvement. The most marked changes were seen in follow-up protocols (r = 0.84), symptom recognition (r = 0.84), and epidemiological knowledge (r = 0.83). By contrast, the objective knowledge items did not retain statistical significance after FDR correction, mainly because baseline performance was already high. Still, knowledge of disease progression associated with diagnostic delay increased from 84.2% to 100%. Conclusions: In this uncontrolled pre–post study, a brief multidisciplinary educational intervention was associated with improvements in primary care physicians’ self-perceived knowledge of STS, whereas no significant changes were detected in objective knowledge after correction for multiple comparisons. Because the study lacked a control group and long-term follow-up, these findings should be regarded as preliminary and require confirmation in controlled studies.

## 1. Introduction

Primary care physicians (PCPs) serve as the first point of contact for most patients presenting with soft tissue masses, placing them in a critical position for early cancer detection [1,2]. However, several studies have consistently demonstrated significant gaps in oncology-related knowledge among PCPs. Backman et al. reported that general practitioners perceived their cancer-related knowledge as insufficient and expressed the need for additional educational support [3]. Similar findings have been described across various oncological settings, including breast, oral, and lung cancer, where physicians identified the need for more practical training and greater involvement in cancer care pathways [4,5,6].

Regarding sarcomas specifically, Fossum et al. demonstrated limited familiarity among PCPs with sarcoma diagnostic guidelines and referral criteria [7]. This is particularly concerning given that soft tissue sarcomas (STS) represent a heterogeneous group of malignant mesenchymal neoplasms with more than 100 histological subtypes, substantial biological complexity, and challenging diagnostic features [8,9]. Although individually rare—with an estimated European incidence of 4–5 cases per 100,000 inhabitants per year—rare cancers collectively account for approximately 20–24% of all cancer diagnoses [10,11]. The management of soft tissue sarcomas has become increasingly complex with the introduction of new treatment strategies. Histology-specific targeted therapies, such as NTRK and ALK inhibitors, together with immunotherapy combinations and epigenetic agents, have broadened the range of available options. At the same time, these advances have reinforced the need for referring physicians to identify patients promptly and facilitate their access to specialized multidisciplinary care [12]. Sarcomas may arise in virtually any anatomical location and frequently present with nonspecific clinical manifestations, making early diagnosis particularly difficult [13]. Diagnostic delay remains common and has been associated with increased tumor size at presentation, more complex surgical management, and decreased overall survival [14,15]. The diagnostic pathway is often prolonged, with patients frequently consulting multiple healthcare providers before a definitive diagnosis is established [16]. Contributing factors include the rarity of sarcomas, their clinical overlap with far more common benign soft tissue lesions, and limited exposure to sarcoma-specific training during medical education [7,17,18].

Current European Society for Medical Oncology—European Reference Network for Rare Adult Solid Cancers—European Reference Network on Genetic Tumour Risk Syndromes (ESMO–EURACAN–GENTURIS) clinical practice guidelines recommend early referral of suspected sarcoma cases to expert multidisciplinary centers, as specialized management has been associated with improved survival and quality of care [9]. Referral criteria typically include the presence of a soft tissue mass larger than 5 cm, deep location, progressive growth, pain, or recurrence after prior excision [19,20,21]. Nevertheless, adherence to these recommendations remains inconsistent, and many patients continue to be managed outside specialized centers, potentially compromising outcomes [10,22].

Educational interventions in primary care have frequently been evaluated using pre-post designs, which are practical in this setting but carry well-known constraints. Threats to internal validity such as testing, maturation, instrumentation and history limit causal inference, and self-reported competence is additionally subject to framing, recall, social desirability and response-shift bias [23,24]. Response shift has been estimated to account for a difference of one to two points on a five-point scale, which can generate ceiling effects and lead to overestimating learning [24]. These considerations justified the decision to assess objective knowledge items alongside self-perceived measures, and they frame the cautious interpretation of our findings.

To address these gaps, educational interventions have been proposed as a strategy to improve early detection and appropriate referral. Gyawali et al. identified substantial educational needs in oncology training among general practitioners and highlighted a strong willingness to participate in structured training programs [25]. Training programs combining structured referral guidelines with targeted education have been associated with improved referral patterns and earlier diagnosis [26]. Systematic reviews have also examined this issue. Cheng et al. found that educational programs on cancer survivorship for primary care providers improve self-reported knowledge and confidence. However, there is still limited evidence that these improvements lead to measurable changes in clinical practice or patient outcomes [27]. Nekhlyudov et al. reached similar conclusions from a different perspective, showing that primary care physicians continue to encounter several obstacles to assuming a greater role in cancer care, including insufficient training, limited consultation time, and uncertainty about how responsibilities should be shared with specialist services [28]. However, the implementation of such initiatives remains limited. Key barriers include high clinical workload, lack of time, insufficient organizational support, and limited methodological training [29,30,31].

The early recognition of sarcoma in primary care remains particularly challenging, and the reasons for this have been documented from several complementary perspectives. Studies examining diagnostic pathways have identified the rarity of the disease, nonspecific or apparently benign initial presentations, limited clinical exposure among general practitioners, initial misdiagnosis, delayed investigation, and difficulties in timely referral to specialized sarcoma centres [17]. Qualitative research involving health professionals, patients and caregivers has further highlighted diagnostic uncertainty, limited experience with sarcoma, and service availability as contributors to prolonged diagnostic intervals [17,18]. Beyond sarcoma specifically, qualitative work conducted across 23 European countries showed that delays in cancer diagnosis emerge from an interplay of patient, clinician and system factors, including high workload, poor continuity of care, unclear referral pathways and cognitive closure on a benign explanation [32]. Recent real-world primary care data reinforce this picture: around four in five patients with sarcoma consult their general practitioner in the six months before diagnosis, with a median of three to four consultations and repeat visits in more than half of cases, a period described as a diagnostic window in which earlier recognition may be possible [33,34]. Taken together, these findings indicate that the problem is not limited to insufficient awareness of a rare tumour, but extends to the organisation of the referral pathway and to the clinician’s ability to distinguish a suspicious mass from the far more common benign lesions. This is where a targeted educational intervention may have the greatest potential impact.

Several scientific societies and healthcare organizations have recently promoted oncology education programs specifically targeted at primary care. In Spain, initiatives such as the Preventive Activities and Health Promotion Program (PAPPS) of the Spanish Society of Family and Community Medicine (semFYC) have developed recommendations and educational activities focused on cancer prevention and early diagnosis [1]. Despite these efforts, evidence specifically evaluating the impact of educational interventions on sarcoma knowledge among PCPs remains scarce.

Given the central role of primary care in cancer diagnosis, the severity and complexity of sarcomas, and the identified gaps in training, there is a clear need for targeted educational strategies. The present study aimed to evaluate the impact of a structured, multidisciplinary educational program on the knowledge of STS among primary care physicians using a quasi-experimental pre–post design.

## 2. Materials and Methods

### 2.1. Study Design

This was a multicenter, single-group quasi-experimental pre-post study conducted without a control group, designed to explore the impact of a structured educational intervention on primary care physicians’ knowledge of soft tissue sarcomas. The absence of a control group was a deliberate design decision and its implications are acknowledged from the outset: without a concurrent comparison group, the observed changes cannot be attributed with certainty to the intervention, since the passage of time, the maturation of participants, increased familiarity with the questionnaire items on repeated administration (testing effect), and the additional attention received by participants (Hawthorne effect) may each contribute to the observed differences.

Three considerations supported the choice of this design. First, the study was conceived as exploratory, with the aim of estimating the magnitude of change and identifying which knowledge domains are most responsive to training, in order to inform the design of an adequately powered controlled trial rather than to establish effectiveness. Second, no validated benchmark exists for sarcoma-specific educational interventions in primary care, which made it difficult to define a meaningful comparator condition. Third, participants were volunteers who had previously expressed an interest in sarcoma-specific training, in a context where no equivalent educational activity is routinely available, so randomly withholding the intervention from part of this group was considered difficult to justify.

To partially mitigate these constraints, the questionnaire included a block of objective knowledge items alongside the items assessing self-perceived competence. These two blocks are sensitive to non-specific effects to different degrees: self-reported competence is more susceptible to social desirability and to the attention received during the intervention, whereas objective items are more exposed to testing effects on repeated administration. The dissociation observed between the two blocks—substantial improvement in self-perceived competence and no significant change in objective knowledge—is therefore not interpreted as evidence that non-specific effects can be excluded, but as an indication that the improvements in the self-perceived domain are unlikely to be explained solely by a generalised tendency of participants to report higher scores after any educational activity. This observation is reported as a secondary and exploratory signal, and it does not replace the need for a controlled design.

### 2.2. Participants and Sampling

Participants were recruited from respondents to a previous cross-sectional survey on knowledge and clinical practices regarding soft tissue sarcomas, who had been asked whether they wished to take part in a subsequent training activity. Sampling was therefore non-random and voluntary: only physicians and Family and Community Medicine residents who explicitly expressed an interest in the training were enrolled. This strategy was chosen because the intervention consisted of an in-person multidisciplinary workshop with limited capacity, which required a defined group of committed participants, and because recruiting from a previous survey permitted the pre-intervention assessment to be conducted under comparable conditions.

The consequences of this approach are acknowledged. Self-selected volunteers are likely to be more motivated and to have a greater baseline interest in oncology than the wider population of primary care physicians, so the sample cannot be assumed to be representative of the profession as a whole. No formal comparison with a reference population of primary care physicians was possible, and the results are therefore presented as describing the change observed within this specific group rather than as estimates applicable to primary care physicians in general.

The target number of participants was not derived from an a priori power calculation. It was determined by the operational conditions of the intervention, specifically the capacity of the workshop and the availability of the teaching team, and should therefore be regarded as a feasibility-oriented target rather than a statistically determined sample size.

### 2.3. Study Population and Setting

The target population consisted of PCPs and Family and Community Medicine residents (MIRs) practicing in Spain.

- **Inclusion criteria:**
  PCPs.
  MIRs.
  Active clinical practice during the study period.
  Participants were drawn from a previously published cross-sectional study on primary care physicians’ knowledge of soft tissue sarcomas [30], including those who expressed interest in the training and enrolled voluntarily.
- **Exclusion criteria:**
  Incomplete pre- or post-training questionnaires.
  Participants who did not complete the training.

### 2.4. Study Variables

Primary outcome variable:

Objective STS knowledge: assessed by total number of correct answers (range, 0–17) before and after the educational intervention.

Secondary outcome variables:

- STS-specific knowledge: Assessed across six domains (epidemiology, clinical presentation, diagnosis, referral criteria, prognosis, and treatment) before and after the educational intervention.
- Self-perceived knowledge of the recognition and referral of suspected STS before and after the educational intervention.
- Independent variables as follows (Table 1):

### 2.5. Educational Intervention

The training was delivered by a multidisciplinary team consisting of a primary care physician, a pathologist, and a medical oncologist, who delivered one session each. The programme was organised in three one-hour blocks, delivered in the order in which the specialties were listed, so that the session structure reflected the diagnostic sequence from initial presentation to histological confirmation and subsequent oncological management.

The workshop combined short didactic presentations with a case-based discussion led jointly by the three specialists. Participants received a printed summary document containing the referral criteria, the recommended diagnostic pathway and a list of key warning signs. No formal assessment of implementation fidelity was conducted (Table 2).

### 2.6. Data Collection Instrument

Objective STS knowledge was assessed using a structured, self-administered questionnaire specifically developed for this study by an expert panel comprising a primary care physician, an oncologist, and a pathologist. The questionnaire underwent expert content review and was pilot-tested with a sample of primary care physicians prior to implementation. No formal psychometric validation of the instrument was conducted. The questionnaire comprised 17 multiple-choice items assessing the following domains of objective STS knowledge (Table 3):

Each item consisted of a multiple-choice question with a single answer and four response options, only one of which was correct. The items were developed based on current clinical practice guidelines and validated educational resources [9,18,19]. The total score ranged from 0 to 17 points, with one point awarded for each correct answer. Higher scores indicated a greater number of correct answers and, consequently, greater knowledge.

The questionnaire was administered in print and electronically via Google Forms immediately before and after the educational training. Responses were automatically recorded and anonymized for analysis.

In addition to the knowledge assessment, sociodemographic and professional data were collected, including:

- Age and sex.
- Professional category (specialist physician or resident).
- Years of clinical experience.
- Type of practice setting (urban, semi-urban, or rural).
- Previous specific training in oncology.
- Self-perceived knowledge of the recognition and referral of suspected STS (5-point Likert scale).

### 2.7. Statistical Analysis

Descriptive statistics were used to summarize participants’ sociodemographic and professional characteristics. Continuous variables were expressed as mean and standard deviation (SD) or median and interquartile range (IQR), as appropriate according to data distribution. Categorical variables were summarized as frequencies and percentages.

To evaluate the impact of the educational intervention on knowledge and self-perceived clinical competence regarding soft tissue sarcomas, a paired pre-post design was used. Data were collected through a structured questionnaire divided into two sections. Block A assessed self-perceived knowledge across 36 clinical topics using a 5-point Likert scale ranging from 1 (“not at all”) to 5 (“very much”). Block B assessed objective knowledge through 17 multiple-choice items, each with a single correct answer. For analysis, responses in Block B were dichotomized as correct (1) or incorrect (0).

Internal consistency was assessed using Cronbach’s alpha for the 36 items of Block A, calculated separately for the pre-intervention (α = 0.956) and post-intervention (α = 0.953) administrations, and using the Kuder-Richardson formula 20 for the 17 dichotomous items of Block B (KR-20 = 0.629 pre-intervention). The post-intervention KR-20 for Block B was negative (−0.119); because this coefficient is a function of inter-item covariances and becomes unstable when item variance approaches zero and the sample is small, it was not interpreted as a substantive property of the block, and objective knowledge is analysed and reported at item level throughout.

Changes between pre- and post-intervention scores were analyzed according to data type. For Block A, paired changes in Likert-scale responses were assessed using the Wilcoxon signed-rank test. Effect sizes were calculated from the standardized test statistic to estimate the magnitude of change, with positive values indicating improvement after the intervention. For Block B, changes in the proportion of correct responses were assessed using McNemar’s test without continuity correction. The strength of association was expressed as an odds ratio (OR), calculated from the proportion of participants who improved relative to those whose responses worsened. Exploratory analyses were also performed to assess whether participants’ age and years of professional experience were associated with post-intervention change. For Block A, Spearman’s rank correlation coefficient was used to examine associations between these variables and individual score differences. For Block B, univariable logistic regression models were initially fitted, with a single predictor per model, to estimate the probability of improvement. Given the limited number of improvement events for several items, these models were considered unstable and are not reported; the interpretation of associations therefore relies on Spearman’s correlations. Sex differences were explored using Mann–Whitney U tests for Block A and Fisher’s exact test for Block B.

Given the number of item-by-item comparisons within each block, *p*-values were adjusted using the Benjamini–Hochberg false discovery rate (FDR) procedure. Adjusted *p*-values below 0.05 were considered statistically significant.

Because the design was paired, with each participant acting as their own control, statistical power for the primary within-subject comparison was greater than would be expected from a sample of this size in an unpaired design. The sample was nonetheless underpowered for the exploratory subgroup analyses and for the item-level comparisons in the objective knowledge block after false discovery rate correction, and results from these analyses are therefore reported as hypothesis-generating only.

All statistical analyses were performed using R software version 4.5.0 (R Foundation for Statistical Computing, Vienna, Austria;r-project.orgData management and graphical visualisation were carried out in the same environment.

### 2.8. Ethical Considerations

Participation was voluntary and anonymous. Before accessing the online questionnaire, participants were provided with an information sheet and gave informed consent electronically (mandatory checkbox). No patient data were collected. Data were processed in accordance with Regulation (EU) 2016/679 (GDPR) and Spanish Organic Law 3/2018 on data protection.

## 3. Results

### 3.1. Participant Characteristics

Forty-two primary care physicians completed both the pre- and post-intervention assessments. Most participants were women (76.2%), with a mean age of 44.3 years (SD = 9.8). The median duration of professional practice was 15 years (IQR: 8–22), and all participants were actively working in primary care during the study period.

### 3.2. Self-Perceived Knowledge

After applying the Benjamini–Hochberg correction for multiple comparisons, all 36 items evaluating self-perceived knowledge showed significant improvement (adjusted *p* < 0.005 for all comparisons). Effect sizes ranged from r = 0.48 to r = 0.84, indicating moderate to large changes across all domains (Table 4).

**Table 4:** Self-perceived knowledge of the recognition and referral of suspected STS before and after the educational intervention.

Item	*p*-Value	*p*-adj (FDR)	Effect Size (r)	% Improved	% Worsened	% Unchanged
Do you know the prevalence of soft tissue tumors (sarcomas)?	<0.0001	<0.0001	0.83	92.11	2.63	5.26
Do you know the mortality rate of soft tissue tumors (sarcomas)?	<0.0001	<0.0001	0.77	86.84	2.63	10.53
Do you know that a lipoma is a soft tissue tumor?	0.0012	0.0013	0.49	42.11	5.26	52.63
Do you know what a sarcoma is?	<0.0001	<0.0001	0.78	84.21	2.63	13.16
Do you know the incidence/prevalence of this tumor?	<0.0001	<0.0001	0.83	89.47	0.00	10.53
Do you know the typical age at diagnosis?	<0.0001	<0.0001	0.79	84.21	2.63	13.16
Do you know that it is usually diagnosed between 0 and 20 years of age?	<0.0001	<0.0001	0.79	81.58	0.00	18.42
Do you know that it is usually diagnosed between 20 and 50 years of age?	<0.0001	<0.0001	0.80	84.21	0.00	15.79
Do you know that it is usually diagnosed between 50 and 80 years of age?	<0.0001	<0.0001	0.77	81.58	2.63	15.79
Do you know that it is usually diagnosed between 80 and 100 years of age?	<0.0001	<0.0001	0.73	71.05	2.63	26.32
Do you know that delayed diagnosis is associated with rapid and severe sarcoma progression?	0.0001	0.0001	0.61	63.16	5.26	31.58
Do you know that sarcoma has a high probability of presenting with metastasis at diagnosis?	<0.0001	<0.0001	0.68	68.42	5.26	26.32
Do you know the prognosis of the disease?	<0.0001	<0.0001	0.82	89.19	0.00	10.81
Do you know the role of molecular biology in soft tissue tumors (massive sequencing)?	<0.0001	<0.0001	0.79	84.21	5.26	10.53
Do you know the signs typically presented by patients with a soft tissue mass?	<0.0001	<0.0001	0.77	81.08	5.41	13.51
Do you know the symptoms typically caused by a soft tissue mass?	<0.0001	<0.0001	0.80	84.21	2.63	13.16
Do you know the signs typically presented by patients with sarcoma?	<0.0001	<0.0001	0.83	89.47	0.00	10.53
Do you know the symptoms typically caused by sarcoma?	<0.0001	<0.0001	0.84	92.11	0.00	7.89
Do you know the red-flag signs of a mass that would raise suspicion of malignancy.	<0.0001	<0.0001	0.83	89.47	0.00	10.53
Do you know the tests required for diagnosis?	<0.0001	<0.0001	0.73	81.58	2.63	15.79
Do you know whether a plain radiograph should be performed?	<0.0001	0.0001	0.62	68.42	5.26	26.32
Do you know whether an ultrasound should be performed?	0.0004	0.0005	0.56	55.26	5.26	39.47
Do you know whether a computed tomography (CT) scan should be performed?	0.0002	0.0002	0.59	60.53	5.26	34.21
Do you know whether magnetic resonance imaging (MRI) should be performed?	<0.0001	<0.0001	0.76	76.32	0.00	23.68
Do you know whether laboratory tests should be performed?	0.0017	0.0017	0.48	52.63	13.16	34.21
Do you know how to interpret the test results?	<0.0001	<0.0001	0.74	76.32	2.63	21.05
Do you know that a specialized soft tissue tumor/sarcoma unit exists for referral?	<0.0001	<0.0001	0.77	78.95	2.63	18.42
Do you know to which hospital department the patient should be referred?	<0.0001	<0.0001	0.75	86.84	7.89	5.26
Do you know whether referral to dermatology is appropriate?	0.0001	0.0001	0.62	78.95	7.89	13.16
Do you know whether referral to orthopedics is appropriate?	<0.0001	<0.0001	0.74	76.32	5.26	18.42
Do you know whether referral to oncology is appropriate?	0.0001	0.0001	0.61	60.53	2.63	36.84
Do you know whether referral to general surgery is appropriate?	<0.0001	<0.0001	0.80	84.21	0.00	15.79
Do you know whether a biopsy should be performed?	0.0003	0.0003	0.58	55.26	2.63	42.11
Do you know the treatment usually prescribed?	<0.0001	<0.0001	0.83	89.47	0.00	10.53
Do you know the follow-up required for these patients?	<0.0001	<0.0001	0.84	94.74	2.63	2.63
Do you know that the family physician should manage symptoms caused by the disease or its treatment?	<0.0001	<0.0001	0.67	68.42	7.89	23.68

*p*-value and *p*-adj: Wilcoxon signed-rank test for paired samples, adjusted by the Benjamini–Hochberg FDR method. Effect size r: computed as z/√N, where z is derived from the normal approximation of the Wilcoxon statistic; positive values indicate improvement post-intervention (|r| ≥ 0.1 small, ≥0.3 medium, ≥0.5 large). % Improved: participants whose score increased; % Worsened: participants whose score decreased; % Unchanged: no change in score. Significance threshold: α = 0.05.

The greatest improvements were observed in areas where baseline knowledge was lowest. Follow-up protocols showed the largest change (r = 0.84), with 94.7% of participants reporting higher confidence after the intervention. Median scores increased from 2 to 4 on the 5-point Likert scale. Similar improvements were found for symptom recognition (r = 0.84; 92.1% of participants improved) and epidemiological knowledge (r = 0.83; 92.1% improved). This distribution of gains, with larger changes in domains of lower baseline knowledge, is also compatible with regression to the mean and cannot by itself be taken as evidence of the specificity of the intervention.

In contrast, changes were smaller for topics with higher baseline scores, suggesting a ceiling effect. Knowledge related to the identification of lipomas showed one of the smallest improvements (r = 0.49), although this item also had the highest baseline median score (Mdn = 4). The item addressing the role of laboratory tests in the diagnostic work-up showed the lowest effect size (r = 0.48).

### 3.3. Objective Knowledge

Internal consistency for this block was moderate before the intervention (KR-20 = 0.629). The post-intervention coefficient was not interpretable (KR-20 = −0.119), a consequence of the near-zero variance of several items after training combined with the limited sample size; results for this block are therefore presented item by item rather than as a composite score. None of the 17 objective knowledge items remained statistically significant after false discovery rate correction (all adjusted *p* > 0.05). Overall, participants demonstrated a high level of knowledge before the intervention, leaving limited room for measurable improvement (Table 5).

**Table 5:** Objective STS knowledge scores before and after the educational intervention.

Item	*p*-Value	*p*-Adj (FDR)	Odds Ratio	% Correct Pre	% Correct Post	% Improved	% Worsened	% Unchanged
What are soft tissue tumors?	0.317	0.462		97.368	100.000	2.632	0.000	97.368
How many types of soft tissue tumors exist?	0.102	0.273	5.000	76.316	86.842	13.158	2.632	84.211
What is the most common soft tissue tumor?	1.000	1.000	1.000	97.368	97.368	2.632	2.632	94.737
What is sarcoma?				100.000	100.000	0.000	0.000	100.000
What is the incidence/prevalence of sarcoma?	0.782	0.834	0.857	65.789	63.158	15.789	18.421	65.789
At what age is it usually diagnosed?	0.366	0.488	0.571	34.211	26.316	10.526	18.421	71.053
If diagnosis is delayed, sarcoma progression is…	0.014	0.114		84.211	100.000	15.789	0.000	84.211
Sarcoma has the potential to metastasize…	0.317	0.462		94.737	97.368	2.632	0.000	97.368
What is the prognosis of the disease?	0.317	0.462		97.368	100.000	2.632	0.000	97.368
Signs and symptoms of soft tissue tumors	0.083	0.266		92.105	100.000	7.895	0.000	92.105
Signs and symptoms of sarcoma	0.014	0.114		81.081	97.297	16.216	0.000	83.784
Red-flag signs suggesting malignancy	0.655	0.806	0.667	92.105	89.474	5.263	7.895	86.842
What tests should be performed for diagnosis?	0.705	0.806	0.750	76.316	73.684	7.895	10.526	81.579
To which department should a patient with suspected sarcoma be referred?	0.058	0.231	0.250	21.053	5.263	5.263	21.053	73.684
Should a biopsy be performed?	0.317	0.462	3.000	92.105	97.368	7.895	2.632	89.474
Should these patients receive follow-up?	0.317	0.462		97.297	100.000	2.703	0.000	97.297
Should the family physician manage symptoms caused by the disease or its treatment?	0.034	0.181	7.000	68.421	84.211	18.421	2.632	78.947

*p*-value and *p*-adj: McNemar’s test without continuity correction, adjusted by the Benjamini–Hochberg FDR method. Odds Ratio: ratio of discordant pairs (improved/worsened). % Correct pre/post: proportion of correct answers before and after the intervention. % Improved: answered incorrectly pre and correctly post; % Worsened: answered correctly pre and incorrectly post; % Unchanged: no change in response. Significance threshold: α = 0.05.

**Figure 1:**
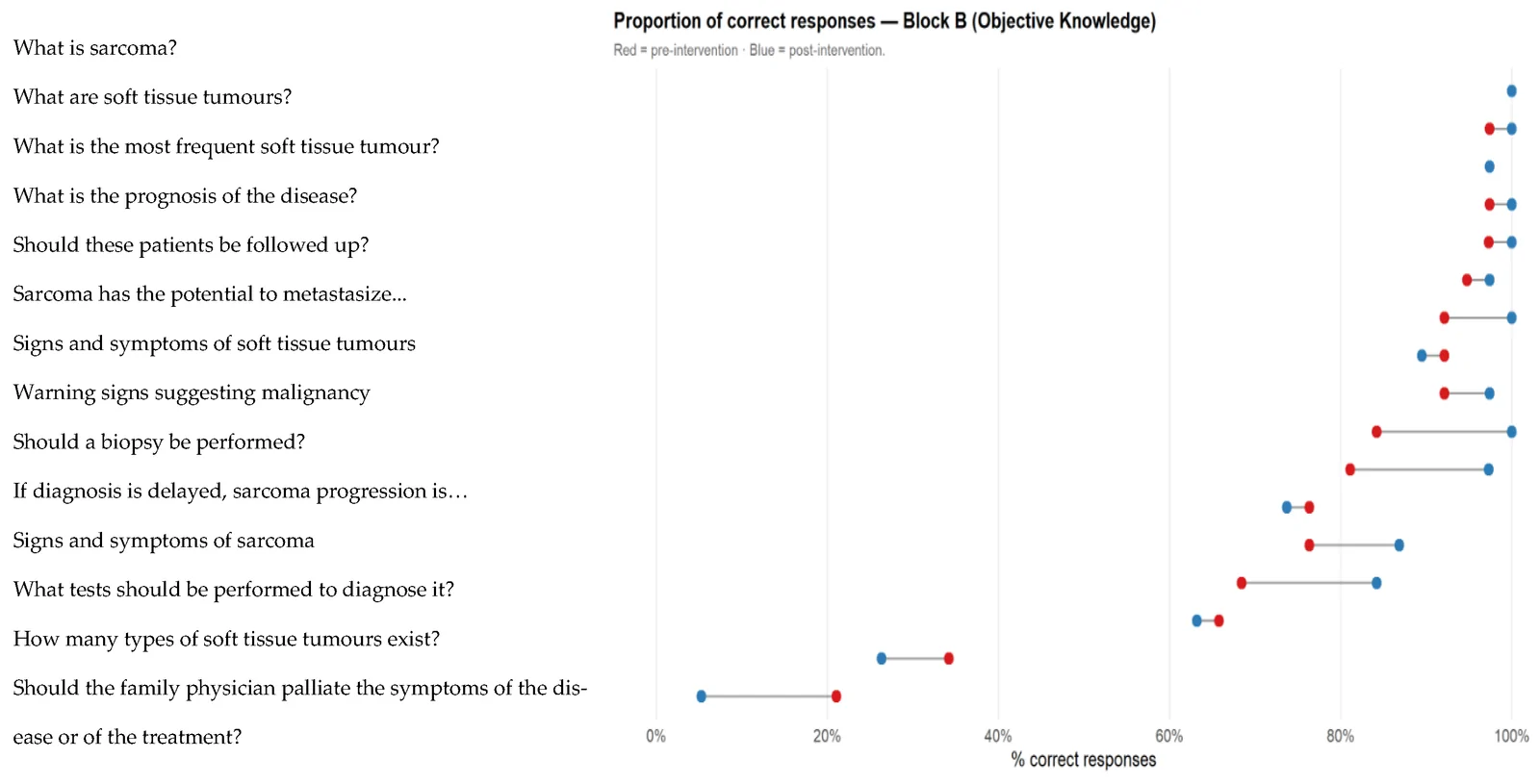
Proportion of correct responses to the Objective STS knowledge assessment before and after the educational intervention. Proportion of correct responses per item before (red) and after (blue) the educational intervention. The connecting segment indicates the direction and magnitude of change. No item reached statistical significance after Benjamini–Hochberg FDR correction (α = 0.05). McNemar’s test without continuity correction was used for all comparisons.

Several questions had very high rates of correct responses at baseline, including the definition of sarcoma (100%), disease prognosis (97.4%), the need for follow-up (97.3%), and biopsy indications (92.1%) (Figure 1). These findings indicate that participants already possessed strong foundational knowledge in several key areas before receiving the educational intervention.

Although no item remained significant after adjustment, two showed clear positive trends. Correct responses regarding disease progression associated with diagnostic delay increased from 84.2% before the intervention to 100% afterwards (*p* = 0.014; adjusted *p* = 0.114). Likewise, recognition of the family physician’s role in symptom management increased from 68.4% to 84.2% (OR = 7.0; 95% CI: 1.2–41.3; *p* = 0.034; adjusted *p* = 0.181).

One unexpected finding was observed for the referral pathway item. The proportion of correct responses to this item fell from 21.1% at baseline to 5.3% after the intervention (OR = 0.25; 95% CI: 0.05–1.19), with 21.1% of participants answering correctly before and incorrectly afterwards. This estimate is based on a small number of discordant pairs and should be interpreted descriptively only.

### 3.4. Exploratory Analyses

No statistically significant associations were found between participants’ sociodemographic characteristics and changes in educational outcomes after correcting for multiple comparisons. Positive correlations were observed between years of professional experience and improvement in several knowledge domains, although none remained significant after adjustment. The strongest associations were found for questions related to sarcoma mortality (ρ = 0.42; *p* = 0.010; adjusted *p* = 0.23) and age-related diagnostic patterns (ρ = 0.36–0.39; *p* < 0.03; adjusted *p* = 0.22–0.24). This pattern is reported as a hypothesis for adequately powered future studies rather than as a substantive finding.

Educational outcomes did not differ significantly between male and female participants. However, this comparison is limited by the small number of male physicians included in the study (*n* = 10).

## 4. Discussion

The present study evaluated the impact of a structured, multidisciplinary educational intervention on primary care physicians’ knowledge of soft tissue sarcomas. To our knowledge, this represents one of the few studies worldwide specifically examining educational interventions targeting sarcoma recognition in the primary care setting, addressing a significant gap in the literature on rare cancer education [7,13,35]. The training program produced substantial improvements in self-perceived knowledge across virtually all domains assessed, with large effect sizes (r = 0.48–0.84) and improvement rates exceeding 80% for most items.

The magnitude of these effect sizes warrants particular consideration. According to Fritz et al. [36], r values of approximately 0.10, 0.30, and 0.50 represent small, medium, and large effects, respectively [36]. In the present study, effect sizes consistently fell within the medium-large to large range, suggesting that the detected improvements are not only statistically reliable but also substantial from an applied perspective. This distinction proves especially pertinent given the challenges inherent to educational research in primary care, where clinically meaningful effects may not always reach conventional significance thresholds due to practical constraints on sample recruitment [28,30].

These findings align with previous research demonstrating that targeted educational interventions can effectively enhance primary care physicians’ confidence in oncological topics [2,25]. Roberts et al. similarly reported significant improvements in provider knowledge and counseling frequency following brief educational interventions in primary care [37]. Systematic reviews have reinforced these observations, concluding that educational programs consistently improve self-perceived knowledge and confidence among primary care providers, although their translation into measurable clinical outcomes remains insufficiently documented [27].

The greatest improvements were observed in areas with the lowest baseline knowledge, particularly follow-up protocols, symptom recognition, and epidemiological aspects, indicating that the intervention effectively addressed knowledge gaps among primary care physicians. This pattern is consistent with evidence suggesting that sarcoma-specific knowledge remains a notable gap in primary care training [7,13]. A recent cross-sectional study conducted in Spain confirmed that awareness of sarcoma warning signs among frontline clinicians remains suboptimal, with limited familiarity with European guideline recommendations despite their perceived usefulness for clinical decision-making [35]. Conversely, items with higher baseline scores, such as lipoma identification and imaging modalities, showed modest improvements attributable to ceiling effects, reflecting pre-existing competency in areas routinely encountered in primary care practice.

In contrast to the subjective domain, objective knowledge items did not reach statistical significance after FDR correction, largely due to high baseline performance. Several items exhibited near-maximal correct response rates before training (e.g., sarcoma definition: 100%; prognosis: 97.4%), precluding meaningful assessment of further improvement. This finding is compatible with the self-selection of participants and should not be interpreted as evidence of the competence of the study population without this qualification; it also limits the capacity of the instrument to detect improvement in this block. Similar ceiling phenomena have been reported in educational studies targeting experienced practitioners [5,6].

It does not follow, however, that no clinically relevant change occurred. Awareness that delayed diagnosis is associated with rapid and severe progression increased from 84.2% to 100%, and recognition of the family physician’s role in symptom management from 68.4% to 84.2%. Neither comparison survived correction, and the first is consistent with a ceiling effect and with limited statistical power. We therefore present these as clinically relevant observations that remain statistically inconclusive rather than as demonstrated effects, and we identify as an explicit question for future research whether equivalent changes would reach significance in a larger sample with a lower baseline level. The clinical relevance of this topic remains important, given evidence that diagnostic delays may adversely affect outcomes and complicate surgical management [14,15].

The correct identification of referral pathways decreased from 21.1% to 5.3%, although this change did not reach statistical significance after correction and should be interpreted descriptively. The reasons for this decline cannot be determined from the data available, since the study included no measure of how participants processed the referral content. A plausible explanation, which would require separate evaluation, is that presenting sarcoma referral across three different disciplines, each with its own perspective and terminology, may leave the destination for a given patient less clear than a single consolidated message would. On this basis, and as a design recommendation rather than as an inference from our results, future programmes should consider delivering referral content as one consolidated block, organised around a single default destination: direct referral of any soft tissue mass meeting warning criteria to the specialised sarcoma unit, without intermediate steps.

Exploratory analyses revealed no significant associations between sociodemographic variables and educational outcomes after multiple comparison correction. Positive correlations between years of experience and improvement in certain items suggest that experienced physicians may derive particular benefit from structured continuing education, consistent with broader observations on professional development in primary care [30]. The recent NCCN Guidelines Insights for Soft Tissue Sarcoma highlight the continuously evolving treatment landscape and recent updates in surgical management and systemic therapy recommendations, reinforcing the importance of ongoing educational updates for clinicians involved in sarcoma care [38].

### 4.1. Strengths and Limitations

This study presents several methodological strengths. The use of a structured pre-post assessment with comprehensive coverage of both subjective and objective knowledge domains allowed for nuanced evaluation of intervention effects. The application of effect size metrics alongside traditional significance testing provides a more complete picture of practical impact, addressing recognized limitations of null hypothesis significance testing in educational research [36]. Furthermore, the systematic correction for multiple comparisons using the Benjamini–Hochberg method enhances the reliability of reported findings. The multidisciplinary nature of the intervention reflects contemporary recommendations for sarcoma education and aligns with ESMO–EURACAN–GENTURIS clinical practice guidelines advocating for integrated care approaches [9].

Certain methodological considerations should be acknowledged when interpreting these results. The sample size (*n* = 42), while consistent with documented recruitment challenges in primary care educational research [29,30], constrained statistical power for subgroup analyses. High baseline performance on objective items produced ceiling effects, which, while limiting detection of further improvement, simultaneously reflects the competency of participating physicians. The single-group design, though common in pilot educational studies, does not permit causal inference; however, it provides essential preliminary data to inform future controlled trials. Self-perceived knowledge, while meaningful for assessing confidence and identifying educational priorities, represents one component of clinical competence that future research should complement with behavioral and patient-level outcomes [3,4].

An important limitation of the present study is the discrepancy between self-perceived and objectively assessed knowledge. Although significant improvements were observed across all self-perceived knowledge items, with moderate-to-large effect sizes, no individual objective knowledge item showed a statistically significant improvement after correction for multiple comparisons. Therefore, improvements in self-perceived knowledge and confidence should not be interpreted as equivalent to improvements in objectively demonstrated knowledge. This discrepancy may partly reflect ceiling effects in some objective items or differences in the sensitivity of the assessment methods. Future studies should incorporate validated objective assessment tools and longitudinal follow-up to determine whether perceived improvements are accompanied by sustained gains in objective knowledge and clinical practice.

The psychometric properties of the instrument are also uneven. The self-perceived competence scale showed high internal consistency (α = 0.956 pre-intervention and 0.953 post-intervention), whereas internal consistency could not be meaningfully estimated for the objective knowledge block after the intervention, given the near-zero variance of several items and the small sample size. Content validity rests on expert consensus without quantified indices, and temporal stability was not assessed, since a repeated administration under unchanged conditions was not possible within the design. The instrument has therefore not undergone full psychometric validation, and such validation—including quantification of content validity and assessment of test–retest stability—should precede its use in confirmatory studies.

**Selection bias and representativeness.** The sample was self-selected. Participants came from respondents to a previous survey who had volunteered for the training, and physicians who choose to attend an educational activity on sarcoma are likely to be more motivated and to hold a greater baseline interest in oncology than their colleagues. The high baseline performance observed in the objective knowledge block is consistent with this possibility and should not be interpreted as evidence of the competence of the study population without this qualification. The group was nevertheless heterogeneous in age, years of professional practice and practice setting, and included both established physicians and residents, which limits but does not eliminate the concern about representativeness. No formal comparison with a reference population was performed, and extrapolation of these results to the broader primary care workforce is not warranted.

**Sample size and statistical power.** The sample size (*n* = 42) was not based on a formal power calculation but on feasibility, reflecting the capacity of the workshop and the practical conditions of recruiting primary care physicians for an in-person activity on a rare tumour. This is a genuine methodological limitation and is stated as such. Two qualifications are relevant for its interpretation. First, the paired design increased power for the primary within-subject comparison relative to an unpaired design of equivalent size; the study was adequately powered to detect effects of the magnitude observed. Second, the study was underpowered for the subgroup analyses and for the item-level comparisons in the objective knowledge block after correction for multiple comparisons, and these findings are presented as exploratory rather than confirmatory. The exploratory analyses of associations with participant characteristics were limited by the number of improvement events per item, which precluded stable modelling; these analyses are reported as hypothesis-generating and no inference is drawn from them. Confirmation of the present results requires a controlled trial with an a priori sample size calculation.

**Intervention duration and standardization.** The intervention was short, lasting three hours, which was the maximum duration compatible with releasing primary care physicians from clinical duties. Knowledge retention was not assessed, so the observed changes describe an immediate post-intervention effect only. Implementation fidelity was not independently verified, and the intervention was delivered by a single teaching team, which should be considered when assessing transferability.

As anticipated in the Methods, the absence of a control group precludes causal attribution, and the divergence between the two assessment blocks should not be read as excluding non-specific effects. The two blocks differ in their susceptibility to such effects, and the objective items may have been affected in the opposite direction to the self-perceived ones. The dissociation is therefore interpreted only as a weak, exploratory signal that the self-perceived gains are unlikely to reflect exclusively a general disposition to report improvement, not as a substitute for a controlled comparison.

### 4.2. Implications

The findings suggest that brief, multidisciplinary training programs can effectively enhance primary care physicians’ confidence in sarcoma recognition. Given the rarity of these tumors, the challenges in early detection, and the documented adverse outcomes associated with diagnostic delay [10,11,17], interventions that heighten clinical awareness may contribute to timelier specialist referral and improved patient outcomes.

The uncertainty observed in referral pathway knowledge, together with the difficulty inherent in sarcoma diagnostic pathways, supports delivering referral content as a single consolidated message in future programmes, organised around a clear default destination and reinforced through algorithmic decision aids or summary reference materials available at the point of care [18,26]. Educational efforts may be most effective when targeting the topics where gaps are most pronounced, informed by a needs assessment carried out before the curriculum is designed [25].

Future research should employ larger samples, controlled designs with randomization, and longer follow-up periods with behavioral and patient-level outcomes to confirm these preliminary findings and assess their translation into clinical practice. Multicenter collaborations may be particularly valuable given the recruitment challenges inherent to rare cancer education research.

## 5. Conclusions

This exploratory, uncontrolled pre-post study suggests that a brief multidisciplinary educational intervention may improve in primary care physicians’ self-perceived knowledge of soft tissue sarcomas. The most pronounced gains occurred in domains with lower baseline familiarity, namely follow-up protocols, symptom recognition, and epidemiological aspects, with large effect sizes (r = 0.48–0.84) and improvement rates exceeding 80% in most items. These effect size estimates are relevant for interpreting results obtained in a modest sample, where statistical significance alone offers limited information about the magnitude of change.

The decline observed in the correct identification of referral pathways following the intervention points to the need for careful instructional design when multidisciplinary content is delivered. Future programs should incorporate simplified referral algorithms, decision aids, and consolidated reference materials to reduce conceptual ambiguity and support decision-making at the point of care.

Ceiling effects identified in objective knowledge items constrained the capacity to detect further improvement, indicating that assessment instruments require population-specific calibration according to baseline competency levels. Given the adverse consequences associated with diagnostic delay in sarcoma, interventions that increase clinical awareness among primary care physicians may contribute to timelier specialist referrals.

Because the study lacked a control group, the sample was self-selected and no a priori sample size calculation was performed, these findings should be regarded as preliminary. Confirmation through studies with larger samples, controlled designs and longitudinal follow-up is required to determine whether the observed change is sustained and whether it translates into modifications in clinical behaviour and patient outcomes.

It should be emphasised, however, these improvements in self-perceived knowledge were not accompanied by statistically significant changes in individual objective knowledge items after correction for multiple comparisons. Because the study lacked a control group, the sample was self-selected and no a priori sample size calculation was performed, these findings should be regarded as preliminary. Confirmation through studies with larger samples, controlled designs and longitudinal follow-up is required to determine whether the observed change is sustained and whether it translates into modifications in clinical behaviour and patient outcomes.

## Figures and Tables

**Table 1:** Sociodemographic and professional variables collected from participants.

Variable	Type	Categories/Units
Age	Continuous	Years
Sex	Categorical	Male/Female/Prefer not to say
Professional category	Categorical	PCP/MIRs
Years of clinical experience	Continuous	Years
Practice setting	Categorical	Urban/Semi-urban/Rural
Previous oncology training	Categorical	Yes/No
Baseline confidence level	Ordinal	1 (very low) to 5 (very high)

**Table 2:** Structure and content of the multidisciplinary educational intervention, organised in three one-hour sessions.

Block	Discipline	Approximate Duration	Content
Epidemiology and clinical presentation	Family physician	~30 min	Incidence and prevalence of soft tissue sarcoma, age at presentation, initial clinical presentation and warning signs
Diagnostic pathway	Family physician/ Pathologist	~45 min	Initial assessment of a soft tissue mass, when to request imaging, role and limitations of biopsy, histological subtypes
Histopathology and molecular biology	Pathologist	~30 min	Diagnostic confirmation, role of molecular biology, common diagnostic pitfalls
Referral criteria and multidisciplinary management	Oncologist	~45 min	Referral criteria, specialised sarcoma units, treatment approach and follow-up
Case-based discussion	All three	~30 min	Interactive review of clinical cases with the three specialists

**Table 3:** Objective STS knowledge domains and their content.

Objective STS Knowledge Domain	Content	Items
Epidemiology	Incidence, prevalence, and demographic distribution of STS	3
Clinical presentation	Alarm signs and symptoms, typical and atypical presentations	6
Diagnosis	Imaging modalities, biopsy indications, and diagnostic pathways	3
Referral criteria	Indications for referral to specialized sarcoma centers according to clinical guidelines	1
Prognosis and management principles	Prognostic factors, general treatment approach, and role of multidisciplinary teams	4

## Data Availability

The original contributions presented in this study are included in the article. Further inquiries can be directed to the corresponding author due to privacy and ethical restrictions.

## Abbreviations

The following abbreviations are used in this manuscript:

STS	Soft Tissue Sarcomas
MIRs	Medicine Internal Residents
PCPs	Primary Care Physicians
ESMO	European Society for Medical Oncology
EURACAN	European Reference Network for Rare Adult Solid Cancers
GENTURIS	European Reference Network on Genetic Tumour Risk Syndromes
PAPPS	Preventive Activities and Health Promotion Program
semFYC	Spanish Society of Family and Community Medicine

## References

[1] López Verde F., Esteva M., Vela Vallespín C. (2016). El médico de familia en la atención al paciente con cáncer. Aten. Prim..

[2] Rubin G., Berendsen A., Crawford S.M., Dommett R., Earle C., Emery J., Fahey T., Grassi L., Grunfeld E., Gupta S. (2015). The expanding role of primary care in cancer control. Lancet Oncol..

[3] Backman C., Johansson U., Hellgren M. (2021). Knowledge of and support for cancer patient pathways among general practitioners and other physicians—A study from Sweden. Scand. J. Prim. Health Care.

[4] Zavaroni M., Oudé-Engberink A., Antoine V. (2021). French general practitioners’ attitude towards breast cancer in older women: A qualitative study. J. Geriatr. Oncol..

[5] Saraswat N., Everett B., Pillay R., Prabhu N., George A. (2020). Knowledge, attitudes and practices of general medical practitioners in developed countries regarding oral cancer: An integrative review. Fam. Pract..

[6] Abeyweera P.D., Brims F.J.H., Piccolo F., Lei C., Manners D. (2021). Australia-wide cross-sectional survey of general practitioners’ knowledge and practice of lung cancer screening. Intern. Med. J..

[7] Fossum C.C., Breen W.G., Sun P.Y., Retzlaff A.A., Okuno S.H. (2020). Familiarity with sarcoma work-up guidelines among primary care practitioners. Mayo Clin. Proc. Innov. Qual. Outcomes.

[8] Fletcher C.D.M., Bridge J.A., Hogendoorn P.C.W., Mertens F. (2013). WHO Classification of Tumours of Soft Tissue and Bone.

[9] Gronchi A., Miah A.B., Dei Tos A.P., Abecassis N., Bajpai J., Bauer S., Biagini R., Bielack S., Blay J.Y., Bolle S. (2021). Soft tissue and visceral sarcomas: ESMO–EURACAN–GENTURIS Clinical Practice Guidelines for diagnosis, treatment and follow-up. Ann. Oncol..

[10] Gatta G., Capocaccia R., Botta L., Mallone S., De Angelis R., Ardanaz E., Comber H., Dimitrova N., Leinonen M.K., Siesling S. (2017). Burden and centralised treatment in Europe of rare tumours: Results of RARECAREnet—A population-based study. Lancet Oncol..

[11] Siegel R.L., Miller K.D., Jemal A. (2017). Cancer statistics, 2017. CA Cancer J. Clin..

[12] Spalato-Ceruso M., El Ghazzi N., Italiano A. (2024). New strategies in soft tissue sarcoma treatment. J. Hematol. Oncol..

[13] Sinha S., Peach A.H. (2010). Diagnosis and management of soft tissue sarcoma. BMJ.

[14] Nakamura T., Matsumine A., Matsubara T., Asanuma K., Uchida A., Sudo A. (2011). The symptom-to-diagnosis delay in soft tissue sarcoma influence the overall survival and the development of distant metastasis. J. Surg. Oncol..

[15] Zagars G.K., Ballo M.T., Pisters P.W.T., Pollock R.E., Patel S.R., Benjamin R.S., Evans H.L. (2003). Prognostic factors for patients with localized soft-tissue sarcoma treated with conservation surgery and radiation therapy. Cancer.

[16] Weaver R., O’Connor M., Carey Smith R., Halkett G.K. (2020). The complexity of diagnosing sarcoma in a timely manner: Perspectives of health professionals, patients, and carers in Australia. BMC Health Serv. Res..

[17] Chotel F., Unnithan A., Chandrasekar C.R., Parot R., Jeys L., Grimer R.J. (2008). Variability in the presentation of synovial sarcoma in children: A plea for greater awareness. J. Bone Jt. Surg. Br..

[18] Hussein R., Smith M.A. (2005). Soft tissue sarcomas: Are current referral guidelines sufficient?. Ann. R. Coll. Surg. Engl..

[19] Dangoor A., Seddon B., Gerrand C., Grimer R., Whelan J., Judson I. (2016). UK guidelines for the management of soft tissue sarcomas. Clin. Sarcoma Res..

[20] Gerrand C., Athanasou N., Brennan B., Grimer R., Judson I., Morland B., Peake D., Seddon B., Whelan J. (2016). UK guidelines for the management of bone sarcomas. Clin. Sarcoma Res..

[21] Lyratzopoulos G., Wardle J., Rubin G. (2014). Rethinking diagnostic delay in cancer: How difficult is the diagnosis?. BMJ.

[22] Harris M., Thulesius H., Neves A.L., Harker S., Koskela T., Petek D., Hoffman R., Brekke M., Buczkowski K., Buono N. (2019). How European primary care practitioners think the timeliness of cancer diagnosis can be improved: A thematic analysis. BMJ Open.

[23] Bylund C.L., Michaels M., Weiss E.S., Patel S., D’agostino T.A., Binz-Scharf M.C., McKee D. (2021). The Impact of an Online Training Program About Cancer Clinical Trials on Primary Care Physicians’ Knowledge, Attitudes and Beliefs, and Behavior. J. Cancer Educ..

[24] Wykowski J.H., Starks H. (2025). What type of self-assessment is best for your educational activity? A review of pre-post, now-then, and post-only designs. J. Gen. Intern. Med..

[25] Gyawali B., Thapa N., Savage C., Carson L.M., Jalink M., Rawal M., Berry S., Poudyal B.S. (2022). Training general practitioners in oncology: A needs assessment survey from Nepal. JCO Glob. Oncol..

[26] Styring E., Billing V., Hartman L., Nilbert M., Seinen J.M., Veurink N., Vult von Steyern F., Rydholm A. (2012). Simple guidelines for efficient referral of soft-tissue sarcomas: A population-based evaluation of adherence to guidelines and referral patterns. J. Bone Jt. Surg. Am..

[27] Chan R.J., Agbejule O.A., Yates P., Emery J., Jefford M., Koczwara B., Hart N.H., Crichton M., Nekhlyudov L. (2022). Outcomes of cancer survivorship education and training for primary care providers: A systematic review. J. Cancer Surviv..

[28] Lawrence R.A., McLoone J.K., Wakefield C.E., Cohn R.J. (2016). Primary Care Physicians’ Perspectives of Their Role in Cancer Care: A Systematic Review. J. Gen. Intern. Med..

[29] Stephenson S., Tang E.Y.H., Tang E., William P., Allan H., Rouse J., Burton M., Wroe C., Bellamy R., Hesselgreaves H. (2022). Barriers and facilitators to primary care research: Views of GP trainees and trainers. BJGP Open.

[30] Rosemann T., Szecsenyi J. (2004). General practitioners’ attitudes towards research in primary care: Qualitative results of a cross-sectional study. BMC Fam. Pract..

[31] Verhoef M.J., Casebeer A.L., Hilsden R.J. (2010). Identifying the barriers to conducting outcomes research in integrative health care clinic settings—A qualitative study. BMC Health Serv. Res..

[32] Hajdarevic S., Högberg C., Marzo-Castillejo M., Siliņa V., Sawicka-Powierza J., Esteva M., Koskela T., Petek D., Contreras-Martos S., Mangione M. (2023). Exploring why European primary care physicians sometimes do not think of, or act on, a possible cancer diagnosis: A qualitative study. BJGP Open.

[33] Holthuis E.I., van der Graaf W.T.A., Drabbe C., van Houdt W.J., Schrage Y.M., Olde Hartman T.C., Uijen A.A., Bos I., Heins M., Husson O. (2024). The prediagnostic general practitioner care of sarcoma patients: A real-world data study. J. Surg. Oncol..

[34] Rafiq M., de Boer J., Mar J., Desai J., Bae S., Gyorki D.E., Di Bella C., Lyratzopoulos G., Lewin J.H., Emery J. (2024). Clinical activity in general practice before sarcoma diagnosis: An Australian cohort study. Br. J. Gen. Pract..

[35] Gracia Rodríguez R., Romero-Rodríguez E., Jimena Medina I., Leiva-Cepas F. (2026). Knowledge and Clinical Practices of Primary Care Physicians Regarding Soft Tissue Sarcomas: A Descriptive Cross-Sectional Study. Healthcare.

[36] Fritz C.O., Morris P.E., Richler J.J. (2012). Effect size estimates: Current use, calculations, and interpretation. J. Exp. Psychol. Gen..

[37] Roberts S., Tompkins B.J., Kennedy A.G. (2022). The effect of an educational intervention on primary care providers’ knowledge, confidence and frequency of patient counselling on strength training and bone density. Musculoskelet. Care.

[38] von Mehren M., Kane J.M., Armstrong S.A., Balach T., Bishop A.J., Buehler D., Carr-Ascher J., Choy E., Cipriano C., Connolly M. (2025). NCCN Guidelines^®^ Insights: Soft Tissue Sarcoma, Version 1.2025. J. Natl. Compr. Cancer Netw..

